# Contemporary use of P2Y12-inhibitors in patients with acute coronary syndrome undergoing percutaneous coronary intervention in Austria: A prospective, multi-centre registry

**DOI:** 10.1371/journal.pone.0179349

**Published:** 2017-06-20

**Authors:** Maximilian Tscharre, Florian Egger, Matthias Machata, Miklos Rohla, Nadia Michael, Manuel Neumayr, Robert Zweiker, Johannes Hajos, Christopher Adlbrecht, Markus Suppan, Wolfgang Helmreich, Bernd Eber, Kurt Huber, Thomas W. Weiss

**Affiliations:** 1 3rd Medical Department with Cardiology and Intensive Care Medicine, Wilhelminenhospital, Vienna, Austria; 2 Department of Cardiology, Medical University Graz, Graz, Austria; 3 4th Medical Department, Cardiology, Hospital Hietzing, Vienna, Austria; 4 Department of Cardiology, Hospital Wels-Griesskirchen, Wels, Austria; 5 Sigmund-Freud University, Medical School, Vienna, Austria; GERMANY

## Abstract

**Background:**

To this day, there is no data concerning guideline adherence on P2Y12-inhibitors in Austria. Prasugrel and ticagrelor have been shown to be superior to clopidogrel in the treatment of acute coronary syndromes (ACS). However, recent data from European registries showed a reluctant prescription policy with rates of clopidogrel at discharge ranging from 35 to 55%.

**Methods:**

In this prospective, multi-centre registry we assessed prescription rates of P2Y12-inhibitors in patients with ACS in four Austrian PCI centres. Parameters associated with the use of clopidogrel have been evaluated in multivariate logistic regression.

**Results:**

Between January and June 2015, 808 patients with ACS undergoing PCI were considered for further analysis. 416 (51.5%) presented with STEMI and 392 (48.5%) with NSTE-ACS. Mean age was 65.7 ± 12.4 and 240 (30.9%) were female. Twenty-eight (3.5%) died during the hospital stay. At discharge, 212 (27.2% of all patients) received clopidogrel, 260 (32.2%) prasugrel and 297 (36.8%) ticagrelor, while 11 (1.4%) did not receive any P2Y12-inhibitor. Of those patients, who were discharged with clopidogrel, 117 (55.2%) had no absolute contraindication against a more potent P2Y12-inhibitor. Diagnosis of NSTE-ACS (p<0.001), COPD (p = 0.049), and age (p<0.001) next to factors contributing to absolute contraindication were positively associated with the use of clopidogrel.

**Conclusions:**

Despite a high level of care, a considerable number of patients were not treated with the more potent P2Y12-inhibitors. Parameters associated with a presumably higher risk of bleeding and side-effects against the more effective P2Y12 inhibitors were the most prominent factors for the prescription of clopidogrel.

## Introduction

Cardiovascular disease is the most common cause of death in the western world, with coronary artery disease making up its greatest proportion. [1] Considerable time and effort has been invested to improve acute and chronic medical treatment with positive effects on various outcome parameters. [2,3] The introduction of two additional oral P2Y12-inhibitors, prasugrel and ticagrelor, extended the armamentarium of this drug class in addition to clopidogrel, and further improved outcome in patients presenting with acute coronary syndrome (ACS).[4,5] Both substances have been adopted into European treatment guidelines, which both recommend the use of these novel P2Y12-inhibitors over clopidogrel for all patients presenting with ACS without overt contraindications.[6–8]

Recent data from different national registries examining the usage of the novel agents in patients with ACS showed a rather reluctant prescription policy, with rates of clopidogrel prescription at discharge ranging from approximately 35 to 55%.[9–14] Hence, a significant percentage of patients presenting with ACS are not treated according to current guidelines in Europe.

In this registry, we sought to assess the prescription rate of the more effective P2Y12-inhibitors prasugrel and ticagrelor in patients presenting with ACS undergoing percutaneous coronary intervention (PCI) and its predictive parameters in four major Austrian tertiary hospitals with acute PCI facilities.

## Methods

Our study complies with the Declaration of Helsinki of 1975, was approved by the local ethics committee (EK-14-220-VK) and informed and written consent has been obtained from all subjects. The “3^rd^ Department of Medicine with Cardiology and Intensive Care Medicine of the Wilhelminenhospital” served as the coordinating and data analysis centre.

### Study population

This prospective, multicentre, observational registry aimed to evaluate the prescription policy of dual antiplatelet therapy in consecutive patients presenting with ACS undergoing PCI in Austria between January and June 2015. Patients were included at four Austrian PCI centres. ACS patients presented either with persistent ST-segment elevation myocardial infarction (STEMI) or non ST-elevation acute coronary syndromes (NSTE-ACS). STEMI and NSTE-ACS were defined and diagnosed according to the current ESC guidelines available at that time.[6–8] According to the respective prescribing information, absolute contraindications against novel P2Y12-inhibitors were: History of stroke or transient ischemic attack (TIA) for prasugrel, history of intracranial haemorrhage for ticagrelor, presence of active bleeding and the indication for a chronic anticoagulation for both agents. [4,5,15] Age above 75 years and body weight below 60 kilograms for prasugrel, and comorbidities linked with severe dyspnea and predisposition for bradyarrhythmia for ticagrelor were defined as relative contraindications. [4,5,16]

### Endpoints

As primary endpoint, the prescription rate of clopidogrel at discharge was investigated. As secondary endpoint, the association of clinical parameters on prescription patterns of clopidogrel were analysed at admission and at hospital discharge.

### Statistical analysis

Data are presented as mean ± standard deviation for normally distributed continuous variables unless depicted otherwise. Continuous variables were compared by the *t*-test or the Mann-Whitney-*U*-test, where appropriate. For the detection of disparities in the distributions of categorical data Pearson’s χ2-test was used.

The association of clinical parameters on the prescription rate at admission and at hospital discharge of clopidogrel was analysed using a binary logistic regression model with step-wise back elimination using a likelihood-ratio test with a *p*-level for entry of 0.05 and a *p*-value for removal of 0.2. When more than two categories were present, dummy variables were introduced to define a reference group. The final models considered the following variables: Clinical presentation (STEMI or NSTE-ACS), age, gender, weight, presence of diabetes mellitus, presence of arterial hypertension, presence of hyperlipidaemia, familiar history of coronary heart disease (CHD), current or prior smoking, history of chronic obstructive pulmonary disease (COPD), site of antiplatelet loading therapy, presence of atrial fibrillation, active bleeding at admission, history of stroke or transient ischemic attack (TIA), history of intracranial haemorrhage, planned operation and peri-interventional anticoagulation treatment regimen regimen (only at discharge). In all statistical tests performed, a two-sided alpha level of *p* < 0.05 was regarded as statistically significant. Statistical analyses were performed using the IBM^®^ SPSS^®^ Statistics 23.0 (IBM Corp., Armonk, USA) software package.

## Results

Between January and June 2015, we enrolled 990 patients presenting with an ACS, of whom 808 (81.6%) underwent PCI with stent implantation, 53 (5.4%) were referred for coronary artery bypass grafting and 129 (13.0%) were treated conservatively. Only patients undergoing PCI were considered for the primary analysis. Of those, 416 (51.5%) presented with STEMI and 392 (48.5%) with NSTE-ACS. Mean age was 65.7 ± 12.4 years and 250 (30.9%) were female. Baseline characteristics are summarised in Table 1.

**Table 1:** Baseline characteristics, comorbidities and treatment stratified for all patients, STEMI and NSTE-ACS.

Variable	All	STEMI	NSTE-ACS	*p-Value*
	n = 808	n = 416	n = 392	
Age, years (mean±SD)	65.7±12.4	64.3±12.4	67.1±12.2	**0.001**
Female, n (%)	250 (30.9%)	132 (31.7%)	118 (30.1%)	0.617
Weight, kg (mean±SD)	82.1 ± 16.9	81.4±16.0	82.8±17.8	0.236
Hyperlipidaemia, n (%)	488 (60.8%)	255 (61.9%)	233 (59.6%)	0.504
Hypertension, n (%)	573 (70.9%)	257 (61.8%)	316 (80.6%)	**<0.001**
Familiar history of CHD, n (%)	56 (7.0%)	32 (7.8%)	24 (6.1%)	0.365
Diabetes mellitus, n (%)	184 (22.8%)	84 (20.2%)	100 (25.5%)	0.072
Current or prior smoking, n (%)	284 (35.4%)	150 (36.4%)	134 (34.3%)	0.527
COPD, n (%)	54 (6.7%)	21 (5.0%)	33 (8.4%)	0.055
Prior stroke or TIA, n (%)	37 (4.6%)	17 (4.1%)	20 (5.1%)	0.490
Intracranial haemorrhage, n (%)	8 (1.0%)	7 (1.7%)	1 (0.3%)	**0.041**
Active bleeding, n (%)	9 (1.1%)	4 (1.0%)	5 (1.3%)	0.671
Atrial fibrillation, n (%)	79 (9.8%)	38 (9.1%)	41 (10.5%)	0.526
Peri-interventional anticoagulation and antiplatelet therapy				
UFH, n (%)	580 (71.8%)	259 (62.3%)	32 (81.9%)	**<0.001**
UFH + GPI, n (%)	102 (12.6%)	78 (18.8%)	24 (6.1%)	
Bivalirudin, n (%)	95 (11.8%)	57 (13.7%)	38 (9.7%)	
Other^a^, n (%)	22 (2.7%)	17 (4.1%)	5 (1.3%)	
Vessel Intervention, n (%)				
1	603 (79.3%)	318 (82.0%)	285 (76.6%)	**0.045**
2	136 (17.9%)	57 (14.7%)	79 (21.2%)	
3	21 (2.8%)	13 (3.4%)	8 (2.2%)	
Use of DES, n (%)	795 (98.4%)	405 (97.4%)	390 (99.4%)	0.178
Site of antiplatelet loading therapy				
Ambulance, n (%)	168 (20.8%)	137 (32.9%)	31 (7.9%)	**<0.001**
Emergency ward, n (%)	181 (22.4%)	109 (26.2%)	72 (18.4%)	
Cardiology ward, n (%)	153 (18.9%)	66 (15.9%)	87 (22.2%)	
Cath lab, n (%)	220 (27.2%)	67 (16.1%)	153 (39.0%)	
Other wards, n (%)	15 (1.9%)	8 (1.9%)	7 (1.8%)	
Maintenance, n (%)	18 (2.2)	2 (0.5%)	16 (4.1%)	

Data are presented as mean ± SD or %. CHD = coronary heart disease; COPD = chronic obstructive pulmonary disease; DES = drug-eluting stent; GPI = glycoprotein IIb/IIIa inhibitor; NSTE-ACS = non-ST-elevation acute coronary syndrome; STEMI = ST-elevation myocardial infarction; UFH = unfractionated heparin.

^a^ Other refers to peri-interventional monotherapies or combinations of low-molecular weight heparins, fondaparinux and glycoprotein inhibitors.

As expected, patients presenting with NSTE-ACS were older (67.1±12.2 vs. 64.3±12.4; *p* = 0.001) and received multi-vessel PCI more frequently compared to STEMI patients (*p* = 0.045). Also, arterial hypertension (80.6% vs. 61.8%; *p*<0.001) was more frequently observed in the NSTE-ACS population. Moreover, STEMI and NSTE-ACS patients differed significantly in regard to peri-interventional anticoagulation and antiplatelet therapy (*p*<0.001) with higher rates of glycoprotein-inhibitors (18.8% vs. 6.1%) and bivalirudin (13.7% vs. 9.7%) in patients presenting with STEMI. There was no significant difference between both patient groups concerning the use of drug-eluting stents (405 (97.4%) vs. 390 (99.4%); *p* = 0.178).

Of all patients, 122 (15.1%) had an indication for chronic anticoagulation or at least one absolute contraindication against the use of a novel agent at admission (Table 1).

### Antiplatelet therapy at admission

**Fig 1:**
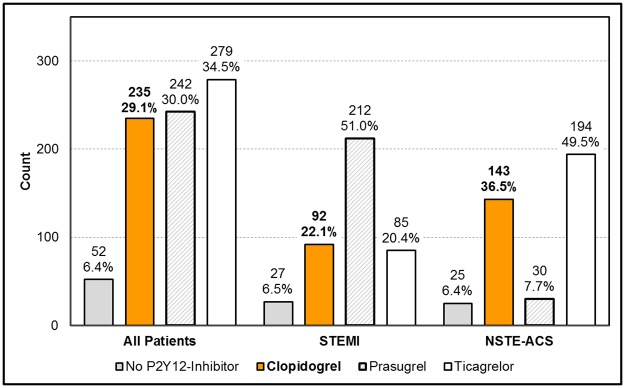
Prescription rates for P2Y12-inhibitors at admission in absolute numbers and percent stratified for the whole cohort, for STEMI patients and NSTE-ACS patients.

Of 808 patients included into the final analysis, 235 (29.1%) received clopidogrel, 242 (30.0%) prasugrel and 279 (34.5%) ticagrelor at admission, while 52 (6.4%) did not receive any P2Y12-inhibitor (Fig 1). In total, 803 patients (99.4%) received acetylsalicylic acid (ASA). Prescription rates of P2Y12-inhibitors for patients presenting with STEMI and NSTE-ACS are also depicted in Fig 1.

The loading dose was given to 168 patients (20.8%) in the ambulance (pre-hospital loading), to 181 (22.4%) in the emergency room, to 153 (18.9%) at the cardiology ward, to 220 (27.2%) in the cath-lab, and to 15 (1.9%) at another ward and 18 (2.2%) were already on a maintenance dose (Table 1).

### Antiplatelet therapy at discharge

**Fig 2:**
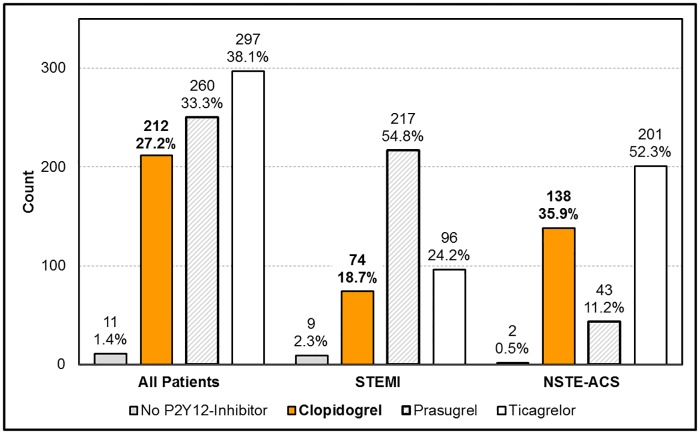
Prescription rates for P2Y12-inhibitors at discharge in absolute numbers and percent stratified for the whole cohort, for STEMI patients and NSTE-ACS patients.

During the hospital stay 28 patients (3.5%) died. Of the remaining patients, 212 (26.2% of all) received clopidogrel, 260 (32.2%) prasugrel and 297 (36.8%) ticagrelor, while 11 (1.4%) did not receive any P2Y12-inhibitor at discharge (Fig 2). Differences between STEMI-patients and NSTE-ACS patients are depicted in Fig 2.

Switching from the initial P2Y12-inhibitor occurred in 14.7% of all patients—64 (7.9% of all) of those patients treated initially with clopidogrel were switched, 25 to prasugrel (3.1%) and 30 to ticagrelor (3.7%), respectively.

Of the 212 patients, who were discharged with clopidogrel, 117 (55.2%—15.2% of all patients) had no absolute contraindication against a new P2Y12-inhibitor. Of all patients discharged with a novel agent, twenty (2.6%) patients were discharged despite the presence of an absolute contraindication or the indication for chronic anticoagulation.

### Predictors of use of clopidogrel

In descending order of relative odds, the presence of following characteristics was associated positively with clopidogrel use at admission: Diagnosis of NSTE-ACS (OR 2.334 [95%CI 1.575–3.460]) and age (OR 1.030 per 1-year increase [95%CI 1.013–1.048]), next to factors contributing to absolute contraindication (Table 2).

**Table 2:** Predictors of clopidogrel use at admission after stepwise backward elimination in a multivariate binary logistic regression analysis model. Variables that were entered into the model prior to elimination were: Main diagnosis, age, gender, weight, presence of diabetes, presence of hypertension, presence of hyperlipidaemia, familiar history of coronary heart disease (CHD), current or prior smoking, history of COPD, site of antiplatelet loading therapy, presence of atrial fibrillation, active bleeding at admission, history of stroke or TIA, history of intracranial haemorrhage and planned operation.

Predictors of clopidogrel use at admission.				
	OR	95% CI		*p*-value
Age	1.030	1.013	1.048	**0.001**
Weight	0.990	0.979	1.002	0.096
NSTE-ACS	2.334	1.575	3.460	**<0.001**
Familiar history of coronary heart disease	1.592	.807	3.144	0.180
Loading in the ambulance	0.383	0.122	1.206	0.101
Loading in the emergency room	0.233	0.074	0.736	**0.013**
Loading at the cath-lab	0.199	0.063	0.622	**0.006**
Loading at the cardiology ward	0.339	0.108	1.068	0.065
Active Bleeding	18.163	2.182	151.202	**0.007**
History of intracranial haemorrhage	8.735	1.434	53.226	**0.019**
History of stroke or TIA	2.411	1.056	5.509	**0.037**
Presence of atrial fibrillation	4.254	2.317	7.811	**<0.001**

OR: odds ratio; CI: confidence interval; CHD = coronary heart disease; COPD = chronic obstructive pulmonary disease; NSTE-ACS = non-ST-elevation acute coronary; syndrome; TIA = transient ischemic attack.

At hospital discharge, the following variables were associated positively with clopidogrel use: Diagnosis of NSTE-ACS (OR 2.197 [95%CI 1.350–3.574]), presence of COPD (OR 2.212 [95%CI 1.037–4.718]), and age (OR per 1-year increase 1.049 [95%CI 1.026–1.072]), next to factors contributing to absolute contraindication (Table 3).

**Table 3:** Predictors of clopidogrel use at discharge after stepwise backward elimination in a multivariate binary logistic regression analysis model. Variables that were entered into the model prior to elimination were: Main diagnosis, age, gender, weight, presence of diabetes, presence of hypertension, presence of hyperlipidaemia, familiar history of coronary heart disease (CHD), current or prior smoking, presence of COPD, site of antiplatelet loading therapy, switching of P2Y12-inhibitor during hospital stay, presence of atrial fibrillation, active bleeding at admission, history of stroke or TIA, history of intracranial haemorrhage, planned surgery and peri-interventional anticoagulation and antiplatelet treatment regimen.

Predictors of clopidogrel use at discharge.				
	OR	95% CI		*p*-value
Age	1.049	1.026	1.072	**<0.001**
Weight	0.991	0.977	1.005	0.185
NSTE-ACS	2.197	1.350	3.574	**0.002**
Hypertension	1.653	0.931	2.934	0.086
Familiar history of coronary heart disease	2.105	0.911	4.861	0.081
Loading in the ambulance	0.174	0.048	0.638	**0.008**
Loading in the emergency room	0.178	0.050	0.641	**0.008**
Loading at the cath-lab	0.170	0.048	0.603	**0.006**
Loading at the cardiology ward	0.172	0.047	0.628	**0.008**
Presence of COPD	2.212	1.037	4.718	**0.040**
Active bleeding	35.197	3.787	327.095	**0.002**
History of intracranial haemorrhage	22.347	3.312	150.761	**0.001**
History of stroke or TIA	4.611	1.759	12.088	**0.002**
Presence of atrial fibrillation	19.785	8.727	44.857	**<0.001**
Peri-interventional unfractionated heparin	0.348	0.087	1.384	0.134
Peri-interventional bivalirudin	0.254	0.058	1.118	0.070
Peri-interventional LMWH	0.132	0.015	1.151	0.067

OR: odds ratio; CI: confidence interval; CHD = coronary heart disease; COPD = chronic obstructive pulmonary disease; LMWH = low-molecular weight heparin; NSTE-ACS = non-ST-elevation acute coronary syndrome; TIA = transient ischemic attack.

## Discussion

The main findings of our study investigating the use of P2Y12-inhibitors in a population of subjects with acute coronary syndrome undergoing invasive revascularization exhibited a clopidogrel use in 29.2% of our patients at admission and in 27.2% at hospital discharge.

In the recent GRAPE registry by Alexopoulos et al. conducted in eight Greek hospitals and a study by Kudaravalli et al. conducted in the urban area around Pittsburgh, rates of clopidogrel upon admission were 67.7% and 80.2%, respectively. [9,13] At discharge, the rate was 38.7% in the GRAPE registry and 55.5% in the Italian EYESHOT registry by De Luca et al., whereas the rate in the study by Kudaravalli et al. remained high with 77.6%.[9,10,13] This prominent gap between the European and American cohorts at discharge reflects the different recommendation policies, with the European guidelines favouring the novel agents in contrast to the American guidelines, leaving the choice of agent to the physicians discretion.[6,17–19]

The clopidogrel prescription rate in our cohort was comparably lower to other recent European registries, both at admission and discharge. [9,10] However, besides this positive trend compared to international data, in our hands 55.2% of the patients discharged with clopidogrel had no absolute contraindication against a modern P2Y12-inhibitor or an indication for long-term antithrombotic therapy. The rate was lower compared to the GRAPE registry, where 76.2% of all patients discharged with clopidogrel had no contraindication against a novel agent, but still 14.4% of our total cohort were “undertreated” in regards to antiplatelet therapy. Potential system related factors might be physician´s inertia, thus, suboptimal guideline adherence, lack of resources, time constraints, and other local factors, as previously reported. [20] Moreover, patient related factors might have had a potential impact on the underuse of more effective antiplatelet agents: Higher age, known as a warning but no absolute contraindication for prasugrel treatment, was independently associated with the use of clopidogrel in our cohort, in line with previous studies showing reduced evidence-based therapy in ACS cohorts of elderly patients and with other registries.[9,10,12,21,22] Following a subgroup analysis of the PLATO-trial current European guidelines advise ticagrelor over clopidogrel also in the elderly. However, the evidence of superiority of ticagrelor compared to clopidogrel is weak and remains doubtful, as elderly patients were strikingly underrepresented in, both, the PLATO and TRITON-TIMI 38 trial. [4–8] Since this population has an especially high risk for bleeding complications, an overestimation of the net clinical benefit in this specific subgroup cannot be excluded.

Also, the presence of COPD was associated with clopidogrel prescription. Physicians seemed to be cautious to use ticagrelor with respect to dyspnoea as a frequent side effect, but in such patients prasugrel would have been an option. [4,5] Accordingly, in our hands the clinical presentation was associated with the use of clopidogrel, as seen also in other registries.[9,10,12,13] NSTE-ACS patients more frequently received clopidogrel compared to STEMI-patients (35.9% vs 18.7%), possibly reflecting the higher rate of comorbidities. [23] Interestingly, body weight was not associated with the prescription of clopidogrel.

Most importantly, antiplatelet therapy administered at the time of admission was infrequently switched during hospitalization (14.7%), even when contraindications against a more effective P2Y12 inhibitor were absent. In the EYESHOT registry the switching rate was comparably low, whereas in the GRAPE registry about one third of the all patients were switched to another agent. The higher switching rate in the GRAPE registry thereby most probably reflects the standard use of clopidogrel in the pre-hospital medical services.[9,10]

Similar to the results of the EYESHOT registry, DAPT was initiated in 26.3% of the NSTE-ACS patients already before PCI, although both, ticagrelor and clopidogrel, were never tested in randomized clinical trials addressing the question of pre-loading in NSTE-ACS before PCI, while prasugrel should not be used before coronary anatomy is known and PCI is performed. [10,24]

### Limitations and strengths

These data are derived from four major tertiary hospitals in predominantly urban areas and might not represent all patients hospitalized for an ACS in rural areas of Austria. Nevertheless, in Austria most rural areas are covered by centralised cath-labs in urban areas and therefore, our registry probably provides a real-life picture of current guideline adherence regarding dual antiplatelet therapy and, therefore, is valuable in assessing the translation of scientific evidence to everyday practice in Central Europe. As the study was planned to address prescription policy of P2Y12-inhibtors in Austria, we did not investigate the clinical outcome for our patients and also the study was not powered to address clinical outcome.

## Conclusion

In opposite to the recommendations of at that time valid ESC guidelines, a considerable number of patients were not treated with the more potent P2Y12-inhibitors in urban Austrian ACS networks, but received clopidogrel despite missing absolute contraindications against prasugrel or ticagrelor. Parameters associated with a presumably higher risk of bleeding and/or comorbidities and expected side-effects against the more effective P2Y12 inhibitors based on warnings (age, co-morbidities associated with dyspnea or bradycardia) were the most prominent factors for the initial prescription of clopidogrel. Moreover, we could demonstrate a relatively low willingness of cardiologists for switching clopidogrel if already initiated to one of the more effective P2Y12-inhibtiors as a topic future improvement.

## Supporting information

S1 Dataset “Contemporary use of P2Y12 in Austria_P ONE_Tscharre_et_al.sav”. Dataset is available from the Figshare database: https://doi.org/10.6084/m9.figshare.4903439.v4.
(SAV)

## Abbreviations

ACS: acute coronary syndrome
CHD: coronary heart disease
CI: confidence interval
COPD: chronic obstructive pulmonary disease
DES: drug-eluting stent
GPI: glycoprotein IIb/IIIa inhibito
LMWH: low-molecular weight heparin
NSTE-ACS: non-ST-elevation myocardial infarction
OR: odds ratio
PCI: percutaneous coronary intervention
STEMI: ST-elevation myocardial infarction
TIA: transient ischemic attack
UFH: unfractionated heparin
